# Supplementary material for: Mortality of native and invasive ladybirds co-infected by ectoparasitic and entomopathogenic fungi
Source: PeerJ. 2020 Nov 4;8:e10110. doi: 10.7717/peerj.10110 (PMC7648450; doi:10.7717/peerj.10110)
Supplement: Table S1 — Numbers for the different replicates (separated by “+”) within each assay. [file peerj-08-10110-s001.docx]

**Mortality of native and invasive ladybirds co-infected by ectoparasitic and entomopathogenic fungi**

Danny Haelewaters, Thomas Hiller, Emily A. Kemp, Paul S. van Wielink, David I. Shapiro-Ilan, M. Catherine Aime, Oldřich Nedvěd, Donald H. Pfister, Ted E. Cottrell

**Table S1.** Overview of number of ladybirds used per treatment. Presented are numbers for the different replicates (separated by “+”) within each assay.

| **Ladybird** | ***H. virescens*** | **Treatment** | **Assay #1** | **Assay #2** | **Assay #3** |
| --- | --- | --- | --- | --- | --- |
| *H. axyridis* | No | Control | 6+6+6 | 10+9+10 | 10+10+10+10 |
|  |  | GHA Bb | 6+6+6 | 10+10+10 | 10+10+10+10 |
|  |  | Native Bb | 6+6+6 | 10+10+10 | 10+10+9+10 |
|  |  | Mb | N/A | N/A | 10+10+10+10 |
|  | Yes | Control | 10+10+10 | 8+8+8 | 10+9+10+10 |
|  |  | GHA Bb | 10+10+10 | 8+8+7 | 10+10+10+10 |
|  |  | Native Bb | 10+10+10 | 8+8+8 | 10+10+10+10 |
|  |  | Mb | N/A | N/A | 10+10+10+10 |
| *O. v-nigrum* | No | Control | 10+10+10 | 10+10+10 | 10+10+10+10 |
|  |  | GHA Bb | 10+10+10 | 10+10+10 | 10+10+10+10 |
|  |  | Native Bb | 10+10+10 | 10+10+10 | 10+9+10+10 |
|  |  | Mb | N/A | N/A | 10+10+10+10 |
|  | Yes | Control | 10+10+10 | 9+9+9 | 10+10+10+10 |
|  |  | GHA Bb | 10+10+9 | 9+9+9 | 10+10+10+10 |
|  |  | Native Bb | 10+10+10 | 9+9+9 | 10+10+10+10 |
|  |  | Mb | N/A | N/A | 10+8+10+10 |
