# Supplementary material for: Mortality of native and invasive ladybirds co-infected by ectoparasitic and entomopathogenic fungi
Source: PeerJ. 2020 Nov 4;8:e10110. doi: 10.7717/peerj.10110 (PMC7648450; doi:10.7717/peerj.10110)
Supplement: Table S3 [file peerj-08-10110-s003.docx]

**Mortality of native and invasive ladybirds co-infected by ectoparasitic and entomopathogenic fungi**

Danny Haelewaters, Thomas Hiller, Emily A. Kemp, Paul S. van Wielink, David I. Shapiro-Ilan, M. Catherine Aime, Oldřich Nedvěd, Donald H. Pfister, Ted E. Cottrell

Table S3. Effects of different treatments on ladybird mortality, with standard error.

| **Ladybird** | ***H. virescens*** | **Treatment** | **Average mortality [%]** | **Std. Error** |
| --- | --- | --- | --- | --- |
| *H. axyridis* | *H. virescens*-negative | Control | 5.6 | 4.14 |
|  |  | GHA Bb | 5.7 | 2.78 |
|  |  | Native Bb | 5.2 | 2.74 |
|  |  | Mb | 22.5 | N/A |
|  | *H. virescens*-positive | Control | 53.6 | 14.21 |
|  |  | GHA Bb | 44.9 | 15.06 |
|  |  | Native Bb | 53.6 | 6.53 |
|  |  | Mb | 60.0 | N/A |
| *O. v-nigrum* | *H. virescens*-negative | Control | 4.1 | 1.84 |
|  |  | GHA Bb | 35.3 | 20.14 |
|  |  | Native Bb | 57.5 | 10.45 |
|  |  | Mb | 60.0 | N/A |
|  | *H. virescens*-positive | Control | 35.7 | 6.91 |
|  |  | GHA Bb | 68.4 | 9.75 |
|  |  | Native Bb | 91.0 | 2.85 |
|  |  | Mb | 97.4 | N/A |
